# 2526: Using the multiphase optimization strategy to engineer an optimized STI preventive intervention among college students

**DOI:** 10.1017/cts.2017.289

**Published:** 2018-05-10

**Authors:** Kari Christine Kugler, Amanda E. Tanner, David L. Wyrick, Jeffrey J. Milroy, Brittany D. Chambers, Alice Ma, Linda M. Collins

**Affiliations:** Penn State Clinical and Translational Science Institute, Hershey, PA, USA

## Abstract

OBJECTIVES/SPECIFIC AIMS: The goal of this study is to develop an effective and efficient STI preventive intervention among college students following the principles and phases of MOST. METHODS/STUDY POPULATION As part of the preparation phase, an explicit conceptual model, drawing heavily on theory and prior research, was used to translate the existing science into 5 candidate intervention components (ie, descriptive norms, injunctive norms, expectancies, perceived benefits of protective behavioral strategies, and self-efficacy). For the optimization phase, in Fall 2016 all first-year students (n=3547) from 4 universities were recruited to participate. Students were randomized to 1 of 32 different experimental conditions that included a combination of the candidate intervention components. Component effectiveness was evaluated using data from an immediate post-intervention survey on respective component mediators (eg, alcohol and sex-related descriptive norms). After a second factorial experiment (Fall 2017), only those intervention components that meet the pre-specified criteria of day ≥0.15 will be included in the optimized intervention. The evaluation phase will evaluate the effectiveness of the optimized STI preventive intervention via a randomized-control trial (Fall 2018). RESULTS/ANTICIPATED RESULTS: Preliminary results from the first factorial experiment suggest that descriptive norms and injunctive norms intervention components were significantly effective in reducing post-intervention perceived alcohol prevalence (β=−0.28, *p*<0.001) and approval of alcohol (β=−0.33, *p*<0.001), and sex-related norms (β=−0.23, *p*<.001). These results, in combination with process data, are being used to inform revisions of the intervention components to be included in a second factorial screening experiment. DISCUSSION/SIGNIFICANCE OF IMPACT: This study demonstrates how an iterative approach to engineering an STI preventive intervention using MOST can affect the behaviors of college students and serve as a foundation for other translational science.

